# The effect of brumation on memory retention

**DOI:** 10.1038/srep40079

**Published:** 2017-01-11

**Authors:** Anna Wilkinson, Anne Hloch, Julia Mueller-Paul, Ludwig Huber

**Affiliations:** 1 School of Life Sciences, University of Lincoln, LN6 7DL, United Kingdom; 2 Department of Cognitive Biology, University of Vienna, Althanstraße 14, 1090 Vienna, Austria; 3 Messerli Research Institute, University of Veterinary Medicine Vienna, Veterinaerplatz 1, 1210 Vienna, Austria

## Abstract

Long-term torpor is an adaptive strategy that allows animals to survive harsh winter conditions. However, the impact that prolonged torpor has on cognitive function is poorly understood. Hibernation causes reduced synaptic activity and experiments with mammals reveal that this can have adverse effects on memories formed prior to hibernation. The impact of brumation, the winter dormancy that is observed in ectotherms, on memory remains unknown. The aim of this study was to examine whether an amphibian, the fire salamander (*Salamandra salamandra*), was able to retain learned spatial information after a period of brumation. Twelve fire salamanders were trained to make a simple spatial discrimination using a T-maze. All subjects learned the initial task. Upon reaching criterion, half of the subjects were placed into brumation for 100 days while the other half served as controls and were maintained under normal conditions. A post-brumation memory retention test revealed that animals from both conditions retained the learned response. Control tests showed that they solved the task using learned information and not olfactory cues. This finding contrasts with much of the mammalian research and suggests that the processes involved in prolonged torpor may have a fundamentally different impact on memory in mammals and amphibians.

Memory retention is essential for survival as it allows an animal to remember information about relevant aspects of its environment such as the location of food, the presence of predators and social relationships^1^. Prolonged periods of torpor, such as hibernation and brumation, that allow animals to survive harsh winter conditions^2^, represent a severe challenge for memory. In mammals, hibernation is characterised by highly reduced brain function^3^ and is accompanied by a loss of 50–65% of synapses^4^. As neuronal functions remain active through regular use^5^, it is likely that the decrease in brain activity may have negative effects on memory retention^6^. To date, the experimental data that has investigated this is ambiguous. There is evidence to support significant loss of some aspects of memory (e.g. 6,7) but also some evidence of retention (e.g. 6,8,9; for a full review please see ref. 10). It is clear that retention is adaptive and therefore should be selected for^8^,^10^, however, the current evidence is far from conclusive.

Surprisingly, the impact of prolonged periods of torpor on memory has never been investigated outside the mammalian class. The processes involved in torpor differ radically between warm- and cold-blooded animals; hibernating mammals regularly rouse from torpidity and enter intervals of sleep (which is thought to have positive effects on brain cell regeneration; 3). In contrast, during brumation, the winter dormancy observed in ectothermic species^11^, animals are dependent upon the temperature of their surroundings^12^ and are forced to stay torpid until temperatures rise^6^,^13^.

Hibernation appears to have a negative impact upon neuronal connections, it causes neuronal dendritic arbors to retract; they then regrow rapidly when the animal returns to normothermia^4^. This large-scale restructuring of neural connections is likely to cause memory loss. It has also been demonstrated that hibernation is responsible for dramatic changes in hippocampal connectivity^14^. The only study to date that has investigated changes in the brain structure of amphibians during brumation showed evidence of apoptotic cell death in the cerebral hemispheres of the frog *Rana esculenta*^15^, suggesting that brumation may have a negative impact upon information retention. Further, there is evidence to show that rapid cooling can prevent memory consolidation in the snail *Lymnaea stagnalis*^16^. The cooling is thought to inhibit *de novo* synthesis of proteins required for long-term memory formation^16^. However, once a long-term memory has been formed, cooling may inhibit enzymatic activity^2^ which in turn could reduce the breakdown of memory and result in better retention.

This study examined the effects of brumation on memory retention in an amphibian, the fire salamander (*Salamandra salamandra*). Fire salamanders, a long-lived species, are territorial as adults and show great site fidelity for many years^17^,^18^. These factors support the idea that maintenance of spatial memory over brumation would be strongly selected for in salamanders. Amphibians have traditionally been considered to have poor cognitive abilities (e.g. 19), however, recent research has revealed impressive behavioural flexibility in this group^20^ and there is now evidence of spatial learning in both frogs^21^ and salamanders^22^. Therefore, in this study fire salamanders were trained to make a spatial discrimination using a T maze. After reaching learning criterion they were placed into one of two treatments; animals in the experimental treatment were placed into brumation for 100 days whilst animals in the control treatment were kept under normal keeping conditions for the same duration. Following the treatment period, salamanders were given a memory test in which they received 6 trials without differential reinforcement.

## Results

### Training

**Figure 1:**
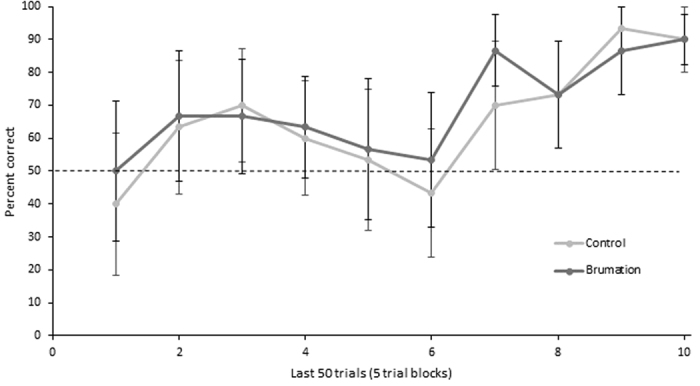
The average percentage correct for last 50 trials of training data for the brumation (n = 6) and control (n = 6) group.

The salamanders learned the task. They took an average of 67.7 (SE ± 3.3) trials to reach criterion and comparison of the first ten and final ten choices showed that they significantly improved over time (t(11) = 7.622, p < 0.001); Fig. 1). There was no significant difference in performance between the brumation and the control group (t(10) = −0.586, p = 0.571) or between rewarded sides (t(10) = −0.586, p = 0.571).

### Memory Test

**Figure 2:**
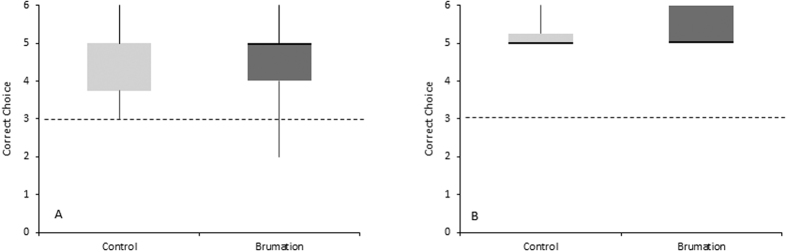
Correct responses in the brumation and control group for (**A**) memory test and (**B**) olfactory test.

The memory retention test revealed no significant difference between the two groups (t(7) = 0.14, p = 0.893), therefore the data were pooled for all further analyses. A Poisson rate test showed that the salamanders performed significantly above chance in the memory test (p < 0.01) suggesting that they had retained the learned response (Fig. 2a).

Any salamander that did not reach an individual score of 5 out of 6 on the memory test was given retraining before the olfactory cue testing. The four re-trained subjects (two control and two brumation group animals) learned significantly faster during retraining (t(3) = 7.836, p = 0.004; average 23.3 SE ± 2.3 trials compared to 67.8 SE ± 4.3 trials) than during the original training.

### Olfactory Test

To ensure that the animals had learned a spatial discrimination and were not simply following the scent of reward, they received additional test trials in which no reward was present and the maze was cleaned thoroughly between each trial. The animals performed significantly above chance in the olfactory test (p = 0.001; Fig. 2b) indicating that they were not using the scent of the reward or other olfactory cues to navigate the maze.

## Discussion

We show that fire salamanders are able to retain learned information over a prolonged period of torpor. This is the first examination of this outside the mammalian clade and suggests that there may be fundamental differences in retention between mammals and amphibians, either caused by the nature of the torpor, or, their learning and memory processes.

The differences in memory retention could be due to key differences between hibernation and brumation. During hibernation warm-blooded mammals generally retain a temperature greater than that of the environment^23^. They regularly increase body temperature, rouse from torpidity and enter intervals of sleep^23^. In contrast, arousal in ectothermic amphibians is dependent upon the temperature of their surroundings^12^. It seems plausible that these differences in body temperature may result in different levels of enzyme activity, which could alter the breakdown of memory.

It is possible that the differences observed are the consequence of differences in the brain regions responsible for spatial processing and/or memory consolidation. The structure of the amphibian brain differs substantially from that of mammals^24^,^25^. Homologous brain regions have been proposed^25^,^26^ but little behavioural work has directly tested the similarities and differences in the spatial learning processes between these groups. Therefore, the mechanisms underlying spatial learning may differ between amphibians and mammals. This could, in turn, affect the way they store and retrieve memories.

The results of this study also lend support to the growing evidence of impressive spatial learning capabilities in amphibians^21^,^22^. All twelve subjects successfully mastered the T-maze and appeared to do so using learned information rather than odour cues. It remains unclear what specific information the animals used. They may have learned spatial information about the specific location of the reward (place learning), as was observed in the frog *Allobates femoralis*^21^, alternatively, they could have learned a motor response (response learning) as was observed in the tiger salamander (*Ambystoma tigrinum*)^22^. Irrelevant of the specific information retained, our findings show strong evidence of impressive long-term memory in the fire salamander. Even those individuals that did not pass the memory retention test performed significantly faster during re-training than during the initial training phase. This is the first evidence to date suggesting that amphibians retain learned memories over a period of months. Taken together, our study revealed that animals from both the brumation and control group retained a learned response for at least 100 days, suggesting that the duration of their long-term memory is extensive and that brumation does not result in forgetting, as has been observed during hibernation in a number of mammalian species.

## Methods

### Animals and apparatus

Twelve captive bred, experimentally naive, one year-old fire salamanders (*Salamandra salamandra*) weighing between 1 and 7 g participated in the study. Due to their age the salamanders’ sex was unknown and it was possible to keep them in groups. They were housed in terrariums (60 × 49.5 × 50 cm) and were maintained at 20 °C (±2 °C), regularly moistened, provided with UV-light (6 am to 6 pm) and fed ad-libitum. The experiment was carried out in accordance with the relevant guidelines and regulations in the country in which it was conducted.

The animals were trained and tested in a T-maze. Each arm contained a small, easily surmountable barrier in front of an opaque box (7.8 × 6 × 2.6 cm). One of the boxes offered a moist hiding place as a reward, while the other could not be entered. The barriers in each arm prevented the animals from seeing which box was accessible. The apparatus was illuminated equally by two lamps and covered with transparent Plexiglas. During the training phase the maze was not cleaned, this was to ensure that it was flooded with odour to discourage the use of olfactory cues.

### Procedure

#### Training

At the onset of each training trial the salamander was placed into the starting area and allowed ten minutes to complete the trial. Upon reaching the goal it was rewarded with three minutes undisturbed access to the box. The trial ended either after the animal had received its reward or after ten minutes had elapsed. First choices were recorded for later analysis. A choice was defined as the salamander touching either barrier with both forelegs. The side of the rewarded arm was counterbalanced between individuals. For the task to be considered learned, each animal had to complete a minimum of 50 trials and get 80% correct first choices during the last 21 trials.

#### Treatment

After the training phase the subjects were separated into one of two treatment groups. In the brumation group, animals were maintained at 4 °C for 100 days (n = 6); in the control group, animals remained under normal keeping conditions for the same period of time (n = 6). After the 100 day treatment period, the animals of the brumation group were returned to their home enclosure and given three days to recover. Then the subjects of both groups were presented with a memory test, which examined retention of the maze task.

#### Memory test

Memory retention test trials were identical to training trials except that no differential feedback was given, animals were removed from the apparatus after touching the barrier. Each salamander received six test trials on three consecutive days without any additional training. Animals that reached an individual score of five out of six correct first choices (80%) passed the memory retention test. Those that failed were re-trained until they reached original performance levels. Sample sizes in training and testing differ due to the loss of three animals during the 100 day treatment period, one from the brumation group and two from the control group.

#### Olfactory test

To ensure that the salamanders had learned spatial information and were not merely going towards the scent of the reward box, all salamanders were given an olfactory test. During this test both boxes were removed and the maze was cleaned thoroughly between trials. Animals received six test trials that were randomly intermixed with training trials. Otherwise, the procedure was identical to that of the memory test. To pass, salamanders needed to reach the criterion of 80% correct first choices. A failure to pass indicated the use of olfactory cues.

## Additional Information

**How to cite this article**: Wilkinson, A. *et al*. The effect of brumation on memory retention. *Sci. Rep.* **7**, 40079; doi: 10.1038/srep40079 (2017).

**Publisher's note:** Springer Nature remains neutral with regard to jurisdictional claims in published maps and institutional affiliations.
